# Anthocyanin accumulation, inflorescence dry weight and total cannabidiol content have different temperature optima in *Cannabis sativa*

**DOI:** 10.1186/s42238-025-00311-w

**Published:** 2025-07-29

**Authors:** Sean R. Kim, Pawan Basnet, Al P. Kovaleski, Shelby L. Ellison

**Affiliations:** 1 Department of Plant and Agroecosystem Sciences, University of Wisconsin- Madison, Madison, WI 53706 USA; 2 Pennsylvania Department of Agriculture, Harrisburg, PA 17110 USA

**Keywords:** Anthocyanin, Cannabis, Temperature effects, Cannabidiol, CBD

## Abstract

**Background:**

Limited information exists on how temperature affects phytocannabinoids and anthocyanin accumulation and inflorescence dry weight yield in *Cannabis sativa*. Understanding how temperature influences these traits is essential for refining cultivation practices, meeting market demands, and developing novel cannabis cultivars with improved agronomic, medicinal, and aesthetic attributes.

**Methods:**

In this study, a day-neutral inbred population with uniform expression of purple pigmentation on the leaves and flowers was used to explore how temperatures ranging from 0.5 to 22 °C impacts inflorescence dry weight, cannabidiol (CBD) percentage, and anthocyanin accumulation in cannabis. Data on inflorescence dry weight (g/plant), CBD (%), and anthocyanin concentration (mg∙L^− 1^) in the primary inflorescence of each plant were collected and analyzed.

**Results:**

Total CBD concentration and inflorescence dry weight yield increased with increasing temperature– likely a result of plant maturity rather than temperature stimuli. Anthocyanin accumulation was significantly affected by temperature stimuli, exhibiting peak production levels at constant temperatures of 8 °C and 15 °C.

**Conclusions:**

CBD concentration and inflorescence dry weight predominantly correlate with plant maturity, whereas anthocyanin accumulation is responsive to variations in environmental temperature. Maximum anthocyanin levels at 8 °C and 15 °C, along with reduction at 0.5 °C and 22 °C, suggests distinct temperature-dependent regulatory pathways for anthocyanin biosynthesis in cannabis, separate from those influencing CBD biosynthesis and inflorescence dry weight. Modeling anthocyanin concentration, CBD concentration, and total inflorescence dry weight across various temperature treatments could optimize desired floral qualities and other traits associated with yield in cannabis.

**Supplementary Information:**

The online version contains supplementary material available at 10.1186/s42238-025-00311-w.

## Introduction

Cannabis (Cannabis sativa) is often cultivated for its secondary metabolites. Yield in cannabis flower production is typically quantified by the weight of harvested inflorescences, harvested trichome heads, or extracted compounds such as phytocannabinoids, flavonoids, terpenes, esters and volatile sulfur compounds (Oswald et al. 2023; Boucher et al. 2025). Cannabis cultivated for the floral market is commonly grown in controlled environments such as hoop houses, greenhouses, and indoor facilities - where quality is often assessed based on the concentration of these bioactive compounds. Optimizing environmental parameters (e.g. photoperiod, light spectrum, temperature, nutrient availability) is a routine practice aimed at improving both yield and phytochemical composition and has been the focus of numerous studies (Bernstein et al. 2019; Kotiranta et al. 2025). While several investigations have examined optimal cultivation conditions for cannabis (Jin et al. 2019), the relationship between temperature stimuli and secondary metabolite production is still poorly understood. Optimal temperature ranges for cannabis growth have been proposed to fall between 25 and 35 °C (Chandra et al. 2011), although this varies based on genotype and developmental stage. Further investigation into how cannabis responds to abiotic stressors, such as low-temperature exposure, is necessary for better understanding plant defense responses and identify opportunities for enhancing the biosynthesis of valuable secondary metabolites.

Among desired secondary metabolites in the cannabis industry, anthocyanins have become notably popular because of their visual appeal, potential protection against environmental stresses, and potential health benefits. Anthocyanins, widely distributed throughout the plant kingdom, are water-soluble compounds that benefit both plant and human health (Khoo et al. 2017; Mattioli et al. 2020). Anthocyanins are a subclass of flavonoids that contribute to the red, blue, and purple pigmentation observed in various plant tissues. Anthocyanins primarily accumulate in the epidermal layers of plant tissues (Chaves et al., 2018) and contribute to plant defense by scavenging reactive oxygen species and mitigating damage from various abiotic and biotic stressors, including ultraviolet (UV) radiation, low temperatures, mechanical injury, herbivory, heavy metal exposure, and nutrient deficiencies (Naing and Kim 2021; Kaur et al. 2023). In addition to their ecological functions, anthocyanins have demonstrated numerous health-promoting effects in humans. High dietary intake of anthocyanins has been associated with improved cardiovascular function (Krga and Milenkovic 2019), antidiabetic activity (Oliveira et al. 2020), neuroprotection against oxidative stress and memory impairment (Pacheco et al. 2018), and nephroprotective effects (Popović et al. 2019). Moreover, anthocyanins exhibit antimicrobial, anticancer, and anti-inflammatory properties (Smeriglio et al. 2016). From an agronomic perspective, increased anthocyanin accumulation in crops has been linked to extended postharvest shelf life and enhanced resistance to pathogenic infections (Bassolino et al. 2013; Zhang et al. 2013).

Beyond their contributions to plant aesthetics, protective functions, human health benefits, and extended shelf life, anthocyanins present an underexplored opportunity in the cannabis industry as natural dyes. In particular, anthocyanin-rich water, a common byproduct of solventless cannabis extraction methods, could be repurposed as a natural, consumable dye with potential functional and nutritional properties. Anthocyanin-based dyes derived from various crops have shown considerable promise as sustainable and health-promoting colorants (Singh et al. 2018; Pramananda et al. 2021). In cannabis, the predominant anthocyanin associated with purple pigmentation in stems, leaves, and floral tissues is cyanidin-3-rutinoside (also known as keracyanin) (Bassolino et al. 2023; Gagalova et al. 2024). This compound exhibits notable bioactivity, including selective cytotoxicity against leukemic cells (Feng et al. 2007), cardiovascular protection in animal models (Thilavech et al. 2017, 2018), enhanced insulin secretion (Kongthitilerd et al. 2022), increased glucose uptake (Choi et al. 2017), and mitigation of high glucose-induced apoptosis (Choi et al. 2018). Optimizing cultivation practices to enhance the accumulation of such compounds requires a clearer understanding of the environmental and physiological factors that regulate anthocyanin biosynthesis in cannabis.

Temperature is a key environmental factor influencing anthocyanin biosynthesis in many plant species. Moderately low temperatures (approximately 12–16 °C) have been shown to enhance anthocyanin accumulation in various crops, likely through the upregulation of transcription factors and structural genes involved in the anthocyanin biosynthetic pathway (He et al. 2020; Jin et al. 2022; Shen et al. 2025). In contrast, elevated temperatures (> 20 °C) are often associated with the downregulation of these regulatory genes, resulting in reduced anthocyanin production (Liu et al. 2019; Leng et al. 2025). Collectively, these findings suggest that cooler temperatures (< 20 °C) promote, while warmer temperatures (> 20 °C) suppress, anthocyanin accumulation across multiple species. However, the relationship between temperature and anthocyanin biosynthesis is complex and influenced by species-specific physiological, genetic, and environmental interactions. In cannabis, the genetic regulation of anthocyanin production remains poorly characterized, although recent studies are beginning to elucidate the underlying mechanisms (Gagalova et al. 2024). Advancing our understanding of how temperature modulates anthocyanin biosynthesis in cannabis will provide valuable insight into species-specific regulatory networks and inform cultivation strategies aimed at enhancing anthocyanin content in both controlled and field environments.

Anthocyanin production in plants is strongly influenced by light. Specific wavelengths, especially blue and ultraviolet (UV) light, can increase the expression of genes involved in anthocyanin biosynthesis (Zoratti et al. 2014; He et al. 2022). In contrast, red light and low-light conditions may reduce anthocyanin levels. The type of light source used—such as LED lighting in controlled environments—can therefore have a strong effect on pigmentation. Light quality not only affects how much anthocyanin is produced, but also significantly changes growth and leaf morphology (Huebner et al. 2024).

Growing degree days (GDD) is a widely utilized metric for estimating plant developmental progress based on the accumulation of heat units over time (Bonhomme 2000; Akyüz and Ransom 2015). It provides a standardized approach for quantifying the influence of temperature on plant growth, making it particularly valuable in controlled environment studies where temperature is the primary manipulated variable. GDD is typically calculated using the following equation: GDD = ((Tmax + Tmin) / 2)– Tbase, where Tmax, and Tmin are daily maximum and minimum air temperatures, and Tbase is the base temperature for a certain species (McMaster and Wilhelm, 1997).

In this study, we explored the effects of temperature on inflorescence dry weight, cannabidiol (CBD) percentage and anthocyanin concentration in cannabis when grown under LED lights. To do so, we exposed plants to a range of temperature treatments applied during flowering, in both constant and fluctuating temperature regimes. The objectives of this study were to quantify how temperature influences anthocyanin concentration, CBD percentage, and inflorescence dry weight in cannabis, and to model these responses under controlled conditions. Understanding these relationships can help optimize production strategies and improve the quality and value of cannabis products.

## Methods

### Plant materials and initial growing conditions

A type III, day-neutral cultivar (Early Harvest 118 S3) with uniform expression of purple pigment on the leaves and flowers was used for all downstream analysis. Early Harvest 118 S3 was self-pollinated for three generations to consistently develop purple pigmentation in both leaves and flowers approximately three weeks after terminal flower initiation, even under warm conditions (25–30 °C), and was selected for its uniform anthocyanin expression at maturity across plant tissues. Plants were grown inside 6.9 × 25.4 centimeter Deepot™ cells (Stuewe & Sons, Inc., USA) using PRO-MIX^®^ HP^®^ MYCORRHIZAE growing medium. For each replication, plants were started from seed at the Walnut Street Greenhouse at the University of Wisconsin–Madison (Madison, WI, USA) and grown for ~ 42 days (or 15 days after terminal flower initiation). All male plants were removed during this process. The first replication was grown from February 20th to April 3rd, 2023, with an average greenhouse temperature of 24.4 °C and an average humidity of 26.8%. The second replication of plants were grown in the greenhouse from April 3rd to May 15th 2023, with an average greenhouse temperature of 25.5 °C and an average humidity of 28.1%. After this initial period of growth, plants showed no sign of purple pigmentation (Figure S1), and female plants were randomly selected and assigned to temperature treatments.

### Experimental design

Temperature treatments were conducted in seven separate Percival^®^ model LT-36VL growth chambers (Percival Scientific, USA) lined with SciWhite LEDs which were separated from chamber growth space by a glass side wall. Growth chambers were monitored hourly throughout the experiments to ensure consistent lighting and temperature. Lighting conditions were set to 515 µmol m⁻² s⁻¹ of light irradiance measured at a distance of 15.24 cm from LEDs, with 16 h of light/8 h dark. The internal space for each growth chamber was 8.5 m^3^ with a total floor area of 3.3 m^2^. Individual plants were randomly distributed in the growth chamber each week to minimize the influence of temperature and light on localized plant response, and each temperature treatment was replicated twice. An experimental unit within each replicate consisted of a single plant grown inside a 6.9 × 25.4 centimeter Deepot™ cell (Stuewe & Sons, Inc., USA), with fifteen plants in replicate one and thirteen plants in replicate two for each growth chamber.

### Treatment 1

The first experiment consisted of 75 plants randomized across five temperature treatments (15 plants per treatment) of 0.5 °C, 4 °C, 8 °C, 15 °C, and 22 °C, each maintained at a photoperiod of 16 h of light and 8 h of darkness for the duration of the experiment. Plants were grown in their respective growth chamber for 30 days before data collection. The temperature treatments were selected to encompass a range of suboptimal temperatures, with 22 °C representing average temperatures experienced by flowering cannabis plants in both field and controlled environments.

### Treatment 2

The second experiment mirrored the first except for 13 plants per treatment and the inclusion of two fluctuating temperature treatments. One fluctuating temperature treatment was 22 °C for 5 h and 4 °C for 19 h, averaging 7.75 °C (hereon referred to as 8 °C fluctuating), while the other was 22 °C for 15 h and 4 °C for 9 h, averaging 15.25 °C (hereon referred to as 15 °C fluctuating). For both fluctuating temperature treatments, the 22 °C period coincided with the 16 h of light.

These fluctuating temperatures were selected to mimic equal GDD as the 8 °C and 15 °C constant treatments. GDD were calculated using a base temperature of 0 °C, with daily GDD determined as the time-weighted average temperature for each 24-hour period. The following equation was used: Daily GDD=(T1​*h1​) + (T2​*h2​) / 24, where T1​ and T2​ are the temperatures during each period, h1​ and h2​ are the number of hours at each temperature, 24 h are in a day, and no subtraction of T_base since T_base = 0 °C. ​Fluctuating temperature treatments were included to investigate the effects of cold temperature stimuli on inflorescence dry weight, CBD percentage, and anthocyanin concentration, while ensuring equal cumulative GDD between treatments. This approach allows for the differentiation of observed effects due to the cold temperature stimulus versus potential limitations in GDD that may prevent the plants from fully maturing and accumulating these compounds. Plants were grown in their respective growth chamber for 30 days before data collection.

### Data collection

From each replicated experiment, total inflorescence dry weight, CBD concentration, and anthocyanin concentration was measured and recorded. To estimate inflorescence dry weight, the top 6 cm of each plant was harvested and placed into flat polyethylene bags before being transferred to a Labconco 700,611,000 Freezone 6 L Floor model Freeze Dryer (Labconco, USA). Samples were freeze-dried for five days, removed from the bags and subsequently weighed. The remaining inflorescences from each plant were dried in a Binder FD 115 heating oven (Binder, Germany) for 5 days at 30 °C and then weighed. The two samples were then combined and the total inflorescence dry weight (g/plant) was determined.

For phytocannabinoid quantification, tissue from the top 6 cm of the primary inflorescence was collected at harvest and processed for total CBD concentration analysis using Ultra Performance Liquid Chromatography (UPLC) by the University of Wisconsin–Madison, Wisconsin Crop Innovation Center (Madison, WI, USA). For each sample, 20 mg of dried, milled tissue was mixed with 1.0 mL extraction solvent (8:2 acetonitrile to methanol) in a 1.5 mL centrifuge tube, containing a ceramic homogenizer, by high-speed shaking at room temperature with a Vortex-Genie 2 Mixer (Scientific Industries, USA) for 10 min. Each sample was then centrifuged for 10 min at 3000 Relative Centrifugal Force (RCF) in an Eppendorf Centrifuge 5910Ri (Eppendorf, Germany) and 125 µL was transferred to an amber microcentrifuge vial containing 875 µL of extraction solvent (a 1:8 dilution). Samples were filtered using a 0.45 μm regenerated cellulose filter vial and run on an Agilent 1290 Infinity II UPLC (Agilent, Germany) using an Agilent Infinity Lab Poroshell 120 EC-C18 3 × 100 mm column (Agilent, Germany) heated at 35 °C. Samples were injected and eluted at a flow rate of 1.0 mL/minute with a changing gradient to match elution: 30:70 water: acetonitrile (0.05% formic acid in both) to 28:72 water: acetonitrile at 1.95 min then an immediate switch to 22:78 water: acetonitrile to 18:82 water: acetonitrile at 4.00 min, ending with 100% acetonitrile at 5.00 min. Absorbance was measured at 220 nm. The following standards were used as calibrants: cannabidiolic acid (CBDA) and CBD (Sigma Aldrich, USA). Total Cannabidiol (CBD) content was calculated as the sum of CBD and (CBDA × 0.877) in accordance with the USDA Hemp Final Rule (U.S. Department of Agriculture 2021).

Anthocyanin content was quantified both visually, and through analytical assays. For the visual analysis, leaf and floral tissue from each plant were visually assessed and were rated: 0 (green/no pigmentation), 1 (lilac/pink), 2 (magenta/reddish), and 3 (purple/dark purple) depending on the shade of the tissue (Figure S2). A total percent anthocyanin coverage of the entire plant was also recorded. These scores were used together to calculate the total anthocyanin score for each plant using the following equation:

Total Anthocyanin Score = (Leaf Score + Flower Score) x Percent Coverage.

For quantification of the total monomeric anthocyanin concentration, the top 6 cm of the primary inflorescence was excised, placed in a plastic bag, and subsequently lyophilized. Once dried, stems were removed, samples were ground into a fine powder, and 0.1 g of ground tissue was weighed into an amber vial. Total anthocyanins were extracted by adding 2 ml of a 2% formic acid in 99.99% methanol solution to each amber vial, which was then left overnight at 3 °C. After the overnight extraction, the amber vials underwent centrifugation to separate particulate matter from the extracted solution. The supernatant from each sample was subjected to a 4-fold dilution using 2% formic acid dissolved in 99.99% methanol before being analyzed via spectroscopy. The total monomeric anthocyanin concentration was determined using the pH differential method (Lee et al. 2005) with some modifications. An aliquot (50ul) of the 4-fold diluted anthocyanin sample was mixed with 150 µl of pH 1.0 buffer (potassium chloride, 0.025 M) and pH 4.5 buffer (sodium acetate, 0.4 M) solutions, respectively, and equilibrated for 30 min at room temperature. An Agilent BioTek 800 TS Absorbance reader (Agilent, USA) was used to measure the absorbance of 96-well plates at 515 nm and 690 nm, using deionized (D.I.) water as a blank. Total monomeric anthocyanin concentration was calculated as cyanidin-3-glucoside equivalents using the following equation:

$$\displaylines{{\text{Anthocyanin pigment (cyanidin}} - 3 - {\text{glucoside equivalents mg}} \cdot {L^{ - 1}}) \cr = \frac{{A\,x\,MW\,x\,DF\,x\,{{10}^3}}}{{\varepsilon xl}} \cr} $$

Where A = (A515nm– A690nm) at pH 1.0– (A515nm– A690nm) at pH 4.5; MW (molecular mass) = 449.2 g/mol for cyanidin-3-glucoside; DF = dilution factor; l = pathlength in cm; ε = 26,900 molar extinction coefficient, in L mol^–1^ cm^–1^, for cyd-3-glu, and 10^3^ = factor for conversion from g to mg. While other monomeric anthocyanins (cyanidin-3-rutinoside, peonidin-3-rutinoside) have been reported in the literature a full characterization was not conducted necessitating the use of a standard reference which is often an indicator of fresh pigment biosynthesis in many species.

### Statistical analysis

All statistical analyses were conducted in R (R 4.1.1) (R Core Team 2021). Reported means in tables and figures represent the means of the two replicated temperature experiments. Individual plants were considered biological replicates within each temperature treatment. The effects of treatments on total CBD (TCBD) were evaluated as CBD percentage on a dry weight basis, total monomeric anthocyanins (TMA) were evaluated as cyanidin-3-glucoside equivalents (mg∙L^− 1^), and inflorescence dry weight (IDW) were evaluated as inflorescence dry weight (g/plant). An outlier test was conducted using Rosner’s generalized extreme studentized deviate test in the EnvStats package, and detected outliers were removed (Millard 2014). Correlation analyses were performed using the Pearson’s correlation test and a 2-tailed significance level of 5%. Data visualization for correlation plots was conducted using the ‘ggplot2’ package (Wickham 2009).

For each response variable, IDW, TCBD, and TMA, a two-way ANOVA was used assess the effect of two factors– average temperature [0.5 °C, 4 °C, 8 °C (including 8 °C fluctuating), 15 °C (including 15 °C fluctuating), 22 °C], condition (fluctuating or constant)– and their interaction. Regressions were analyzed as linear-mixed-effect models using the lmer function in lme4 (Bates et al. 2015). When effects were found to be significant, pairwise comparisons for the slope coefficients were run using Tukey’s Honestly Significant Difference test (lsmeans, emmeans package; Lenth et al. 2022).

Both IDW and TCBD were analyzed with a generalized linear-mixed effect model with temperature as a fixed effect and replication as a random effect. Regressions for IDW and TCBD were analyzed using the lme model in the ‘car’ package (Fox and Weisberg 2019). A one-way ANOVA was used to assess the overall significance of temperature on IDW and TCBD while accounting for the variability introduced by the random effect of replication. The anova function “aov()” in R was used to conduct hypothesis tests for the coefficients in the IDW and TCBD models.

One-way ANOVA was used to analyze the effect of temperature on TMA. Temperature and Temperature^2^ (representing temperature squared values) were treated as continuous predictors in the model. Incorporating Temperature^2^ in the model addresses the observed nonlinear effects of anthocyanin accumulation in response to temperature, ensuring accurate analysis and correction for temperature dynamics. Condition is a binary variable, representing whether the environmental temperature was constant or fluctuating. For each ANOVA, the significance level was set to 5%. If the ANOVA showed significance between treatments, pairwise comparisons for treatment means were run using a Tukey’s post-hoc test(*p* ≤ 0.05).

## Results

### The effect of temperature on inflorescence dry weight

**Fig. 1:**
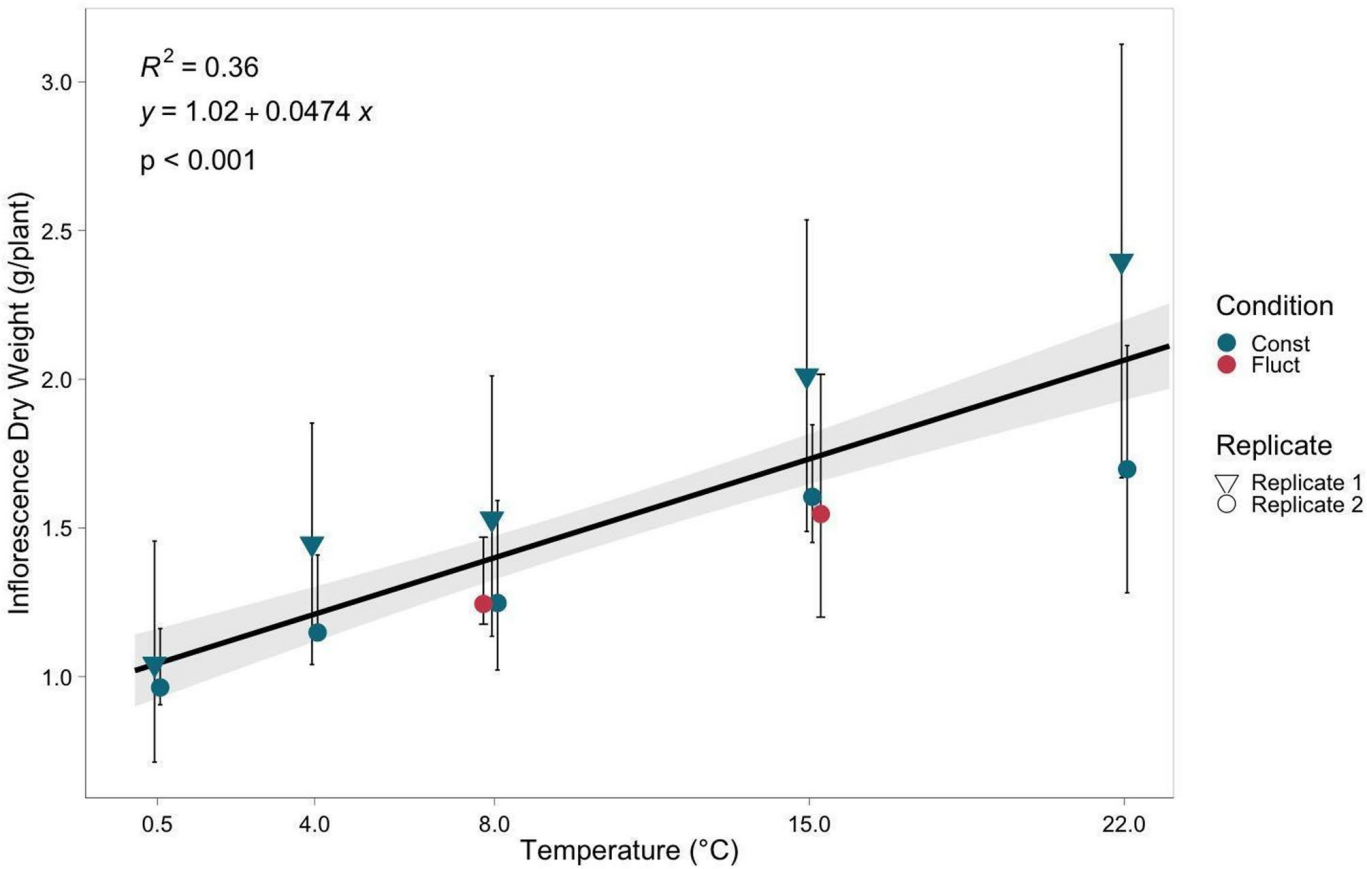
Inflorescence dry weight (IDW) observed for five temperature treatments across two replicated experiments (1 and 2). The trend line represents the linear relationship between temperature and IDW. Error bars indicate the standard deviation of IDW at different temperatures, while grey shading indicates the confidence interval (95%) for the linear regression line

Average inflorescence dry weight (IDW) ranged from 1.0 g to 2.1 g per plant, with the 0.5 °C treatment resulting in the lowest dry weight and the 22 °C treatment resulting in the highest dry weight (Fig. 1). Temperature had a significant positive effect on inflorescence dry weight (*p* < 0.001) (Table 1), with an increase of 0.05 g of inflorescence dry weight per degree increase in temperature within the tested range of temperatures. Plants exposed to fluctuating temperature treatments exhibited no significant differences in IDW when compared to plants exposed to equivalent constant temperature conditions of 8 °C and 15 °C (Fig. 1). Cumulative growing degree days (GDD) were held constant between fluctuating and constant temperature treatments, indicating that differences in IDW were not attributable to variation in total heat accumulation.

**Table 1:** Summary of analysis of variance (ANOVA) results evaluating the effects of temperature, temperature^2^ (temperature squared values to address the observed nonlinear effects of anthocyanin accumulation in response to temperature), condition (constant or fluctuating), and the interaction of temperature and condition on inflorescence dry weight (IDW), total CBD (TCBD), total monomeric anthocyanins (TMA), and total anthocyanin score (TAS)

	*P*-value			
Effect	IDW	TCBD	TMA	TAS
Temperature	***	***	***	***
Temperature^2^	-	-	***	***
Condition	0.9340	0.8834	***	***
Temperature: Condition	0.7746	0.9483	0.4688	0.2624

### The effect of temperature on Cannabidiol percentage

**Fig. 2:**
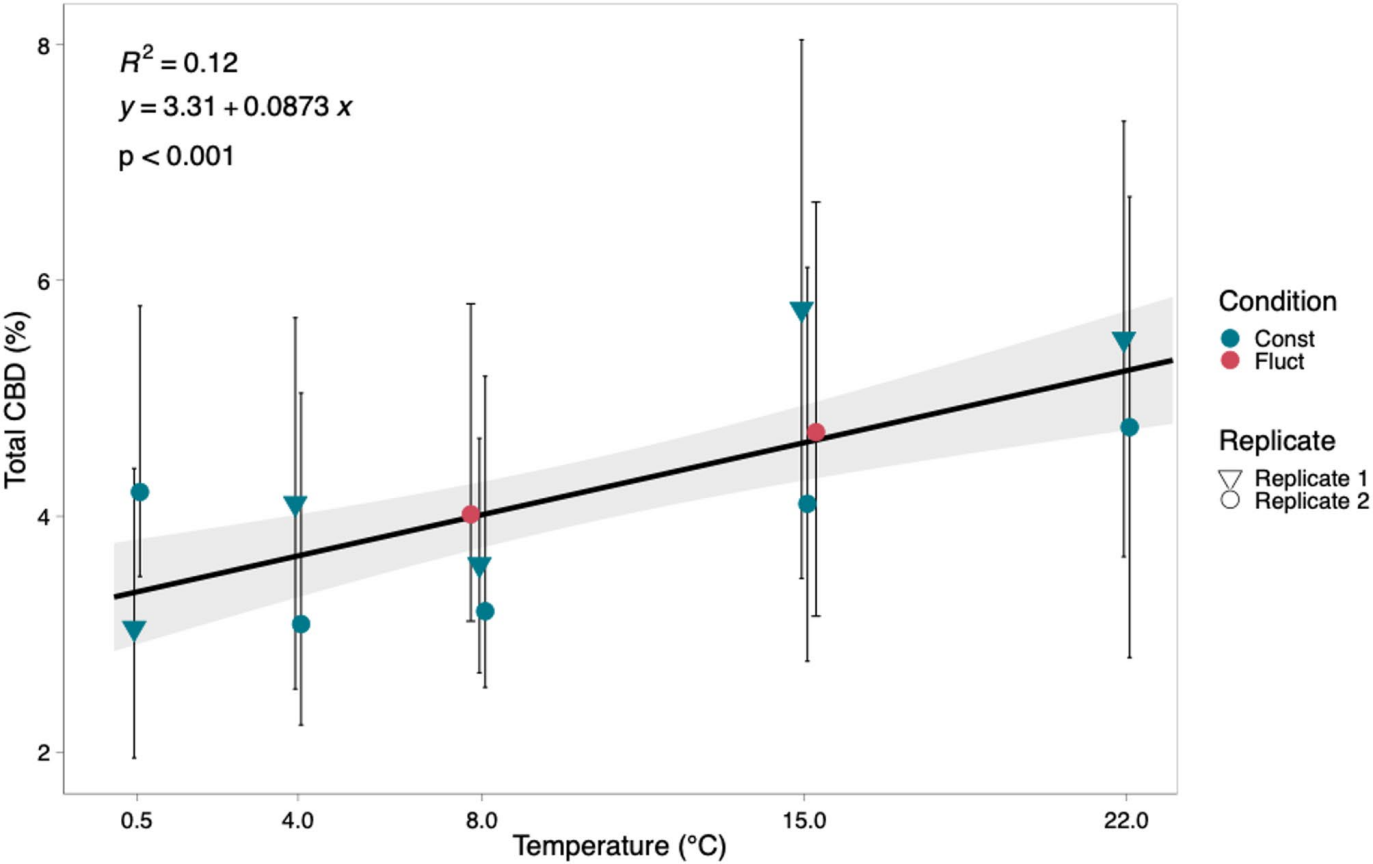
Total CBD (TCBD), measured as a percent dry mass, observed for five temperature treatments across two replicated experiments (1 and 2). The trend line shows the relationship between temperature and TCBD. Error bars indicate the standard deviation for TCBD at different temperatures, while grey shading indicates the confidence interval (95%) for the linear regression line

Average total CBD (TCBD) concentration ranged from 3.4 to 5.2% per plant with the 8 °C treatment resulting in the lowest concentration of TCBD and the 22 °C treatment resulting in the highest concentration (Fig. 2). TCBD concentration was significantly affected by temperature (*p* < 0.001) (Table 1). Plants exposed to fluctuating temperature treatments with an average temperature of 8 °C and 15 °C displayed total TCBD concentration comparable to those recorded in plants cultivated under constant temperature conditions of 8 °C and 15 °C (Fig. 2). TCBD exhibited a positive correlation with increasing temperatures ranging from 0.5 °C to 22 °C and there was an observed increase of 0.09% in total CBD with every degree C increase in temperature. Cumulative growing degree days (GDD) were held constant across fluctuating and constant temperature treatments, suggesting that changes in CBD concentration were driven by thermal exposure rather than differences in total heat accumulation.

### The effect of temperature on anthocyanins

**Fig. 3:**
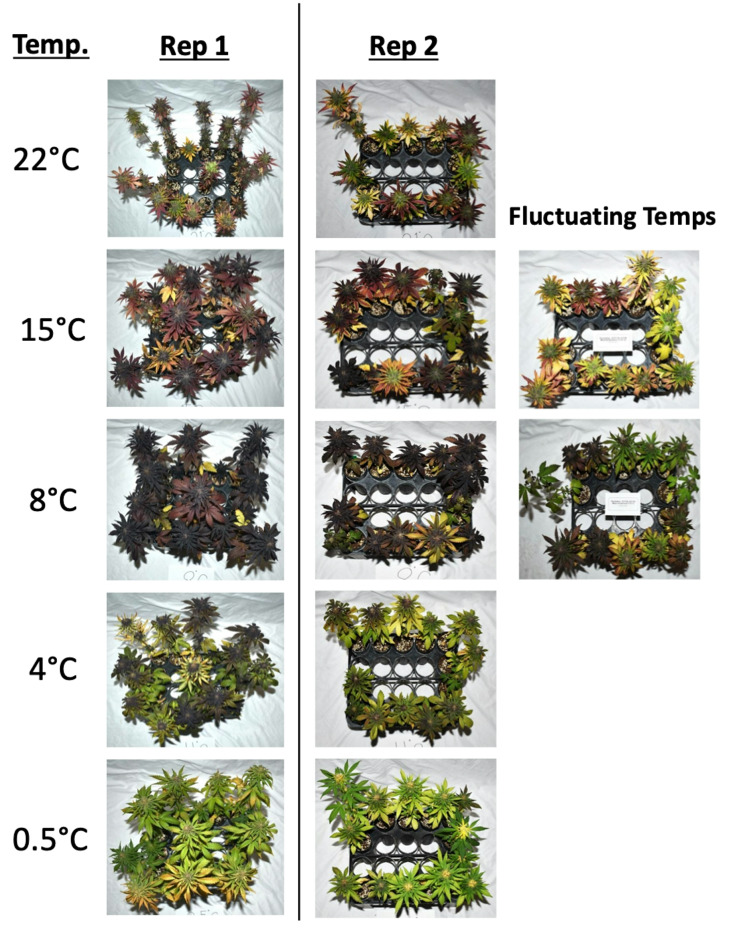
Photographs of plants after their respective temperature treatments. Plants were removed from their growth chambers and photographed 23 and 30 days into their treatments, respectively for replicates 1 and 2. Each photo represents 13-15 plants

**Fig. 4:**
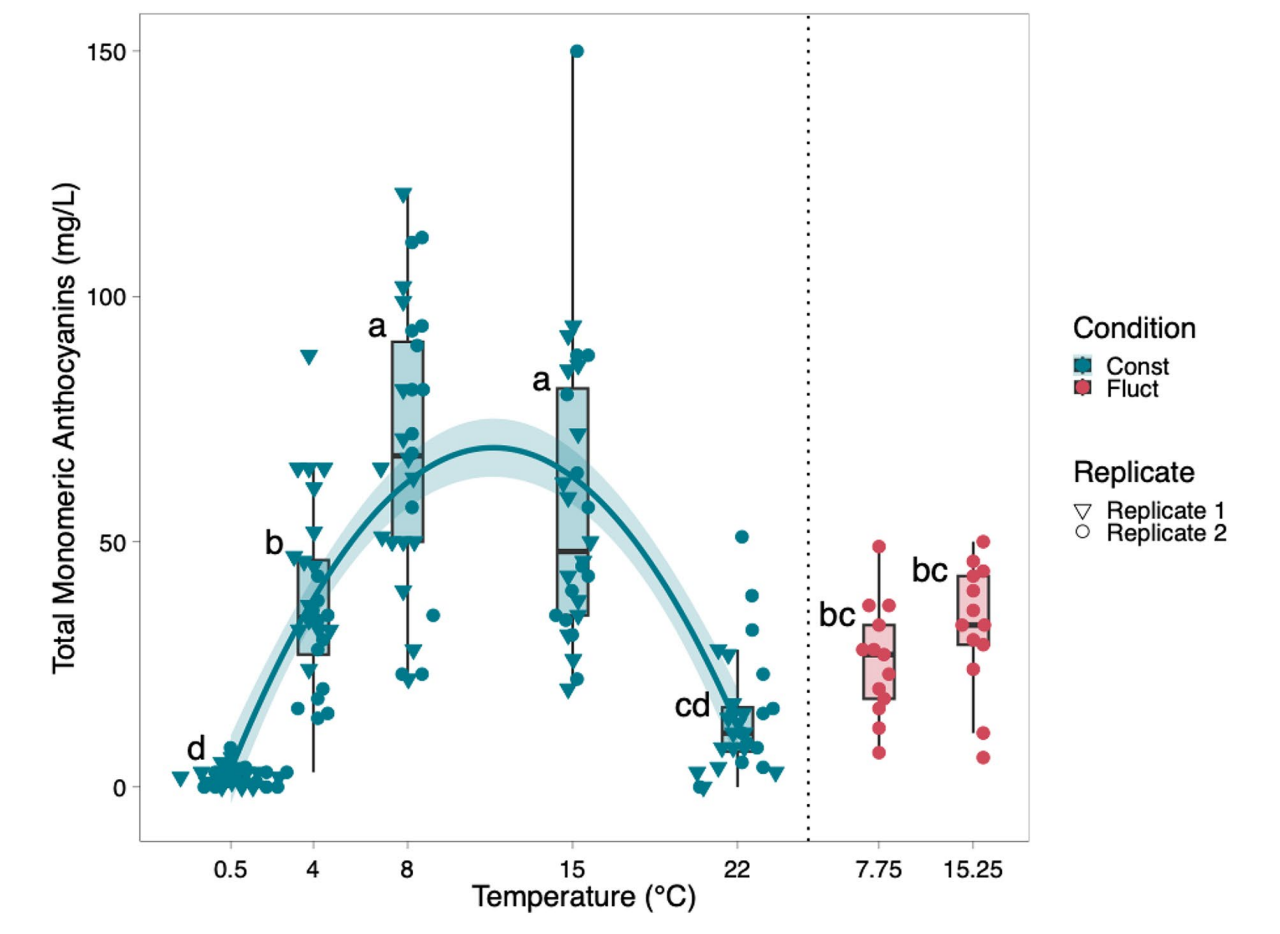
Total Monomeric Anthocyanins (TMA) observed across five temperature treatments and two replicated experiments (1 and 2). Boxplots display the distribution of TMA levels among individuals exposed to various temperature treatments. Boxplots are colored based on experimental conditions (constant or fluctuating). Letters above the boxplots indicate whether treatments were significantly different from each other (*p* < 0.05). Significance was determined using a post hoc Tukey’s HSD test following one-way ANOVA. The trend line represents the quadratic relationship between temperature and TMA while the light blue shading represents the confidence interval (95%) for the trendline

Similar effects were obtained through visual analysis and analytical determinations of anthocyanins (Fig. 3). Plants grown in constant temperatures of 8 °C and 15 °C expressed the highest levels of purple pigmentation with scores of 534 and 533 respectively, while plants subject to a constant 0.5 °C temperature expressed the lowest amount of pigmentation with an average score of 19 (Table S2). The visual rating for anthocyanin accumulation was significantly influenced by the temperature treatments (*p* < 0.001) (Table 1). Both temperature and fluctuating conditions had a significant effect on visual pigment scores (*p <* 0.001) (Table 1). Because cumulative growing degree days (GDD) were matched between fluctuating and constant temperature treatments, the differences in anthocyanin concentration are attributed to the thermal fluctuation itself rather than discrepancies in total heat accumulation. Total monomeric anthocyanin (TMA) levels ranged from 0 mg∙L^− 1^ to 150 mg∙L^− 1^. Plants grown at constant temperatures of 8 °C and 15 °C had the highest levels of total monomeric anthocyanins with an average of 68 mg∙L^− 1^ and 58 mg∙L^− 1^ respectively (Figs. 3 and 4). Plants grown in constant temperatures of 0.5 °C and 22 °C accumulated the lowest quanity of anthocyanins when compared to all other treatments. Plants grown in fluctuating temperature treatments averaging 8 °C and 15 °C, were significantly different from the 0.5 °C, 8 °C and 15 °C treatments when comparing total monomeric anthocyanin levels (Fig. 4). The prediction model that was derived for total monomeric anthocyanins is as follows:

$$\:TMA=\:-2.7+12.33\left(Temperature\right)-$$

$$\:0.528\left(Temperatur{e}^{2}\right)-\:32.53\left(Fluctuation\right)$$

where *Fluctuation* is a binary term of value 1 for fluctuating temperature treatments, and 0 for constant temperature treatments.

### Correlations between traits

**Fig. 5:**
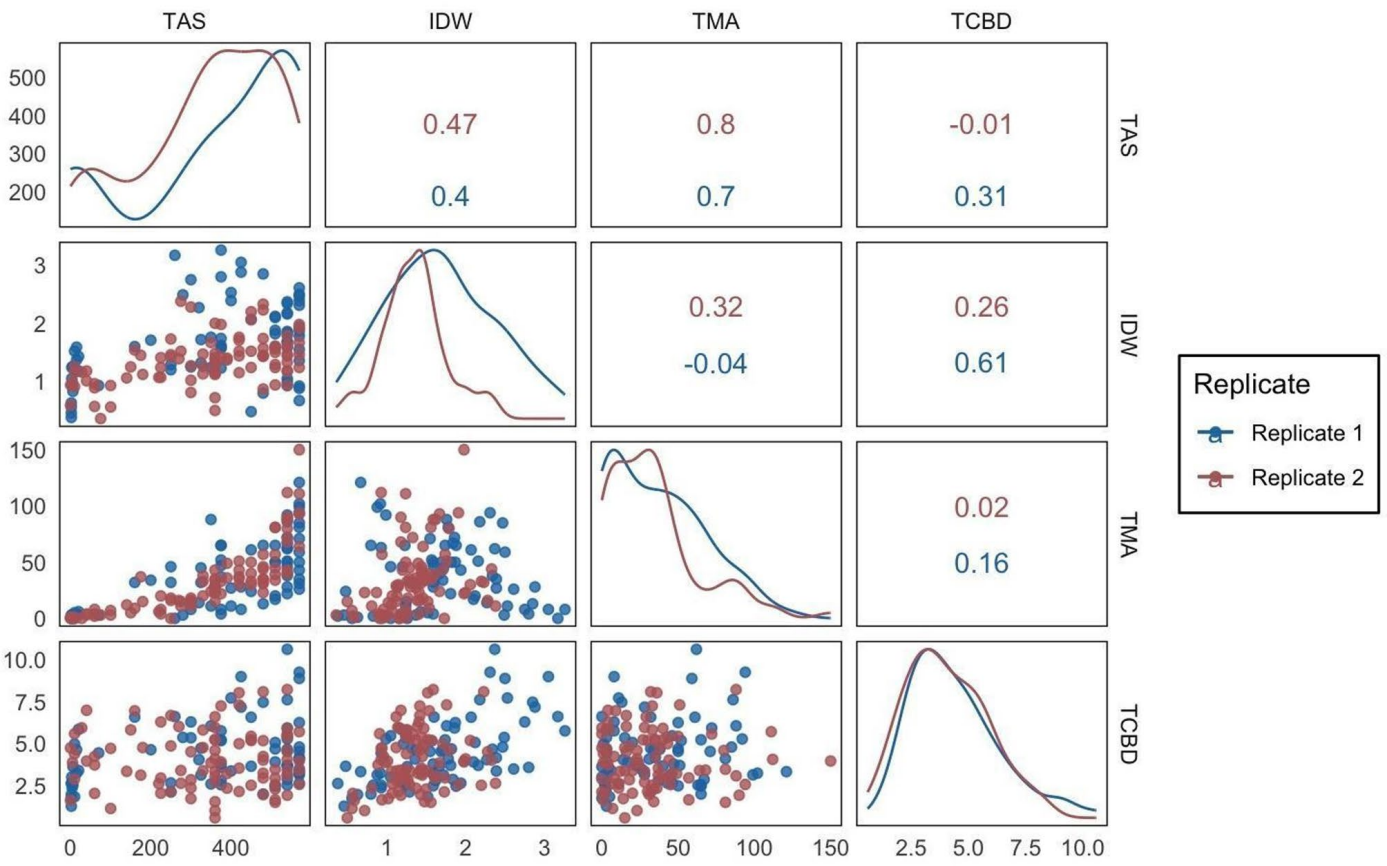
. Correlation between traits for two replicated temperature treatment experiments. IDW: Total Dry Weight (g/plant), TAS: Total Anthocyanin Score, TMA: Total Monomeric Anthocyanins (mg∙L^− 1^), TCBD: Total CBD (%). Significant positive correlations were observed for TAS and TMA (*p* < 0.001), TAS and IDW (*p* < 0.001), and TCBD and IDW (*p* < 0.05)

A strong correlation was found between our visual score (TAS) and spectroscopy results (TMA) in both replications (Fig. 5). A strong positive correlation was observed (0.7 and 0.8, respectively) between total anthocyanin score (TAS) and total monomeric anthocyanins (TMA) for replicate 1 and replicate 2 (*p* < 0.001) (Fig. 5). A positive correlation between TAS and IDW was observed in replicate 1 and 2 with correlation coefficients of 0.40 and 0.47 respectively (*p* < 0.001) (Fig. 5). A positive correlation between TCBD and IDW was observed in replicate 1 and 2 with correlation coefficients of 0.61 (*p* < 0.001) and 0.26 (*p* < 0.05) respectively (Fig. 5). No other correlations were found when comparing IDW, TCBD, TMA, and TAS (Fig. 5).

## Discussion

### Growing degree days influence inflorescence dry weight and Cannabidiol content but not anthocyanins in the inflorescence

In our study, temperature increases, in a range from 0.5 °C to 22 °C, had a positive influence on both dry weight and CBD percentages in the inflorescence. Field observations suggest that growing degree days (GDD) has a significant positive effect on CBD concentration in industrial hemp (Sikora et al. 2011). Our observations align with these findings and suggest that cold temperature stimuli do not significantly affect dry weight or CBD concentration, however, GDD positively influences these traits. When comparing plants subjected to fluctuating temperature treatments to constant temperature treatments with equivalent GDD, no significant differences in CBD percentages were observed (Fig. 2), suggesting that the cold temperature stimulus of 4 °C did not influence CBD synthesis. A similar trend was observed with inflorescence dry weight, and plants exposed to fluctuating temperature treatments exhibited no significant differences compared to those cultivated under constant temperature conditions (Fig. 1). These findings suggest that plant growth and maturity play an important role in determining CBD percentage and inflorescence dry weight in cannabis, rather than signaling from cold temperature stimuli. These findings are consistent with previous research showing that trichome number increases with plant age (Punja et al. 2023). Given that both THC and CBD are synthesized in glandular trichomes, the observed increase in CBD concentration with plant maturity in our study may similarly reflect age-related increases in trichome density. Based on our analysis, we expect that individuals from each treatment would likely exhibit no significant differences in total CBD percentages and inflorescence dry weight if equal GDD was achieved within each temperature treatment.

### Distinct temperature responses for anthocyanin accumulation, inflorescence dry weight, and Cannabidiol accumulation

**Fig. 6:**
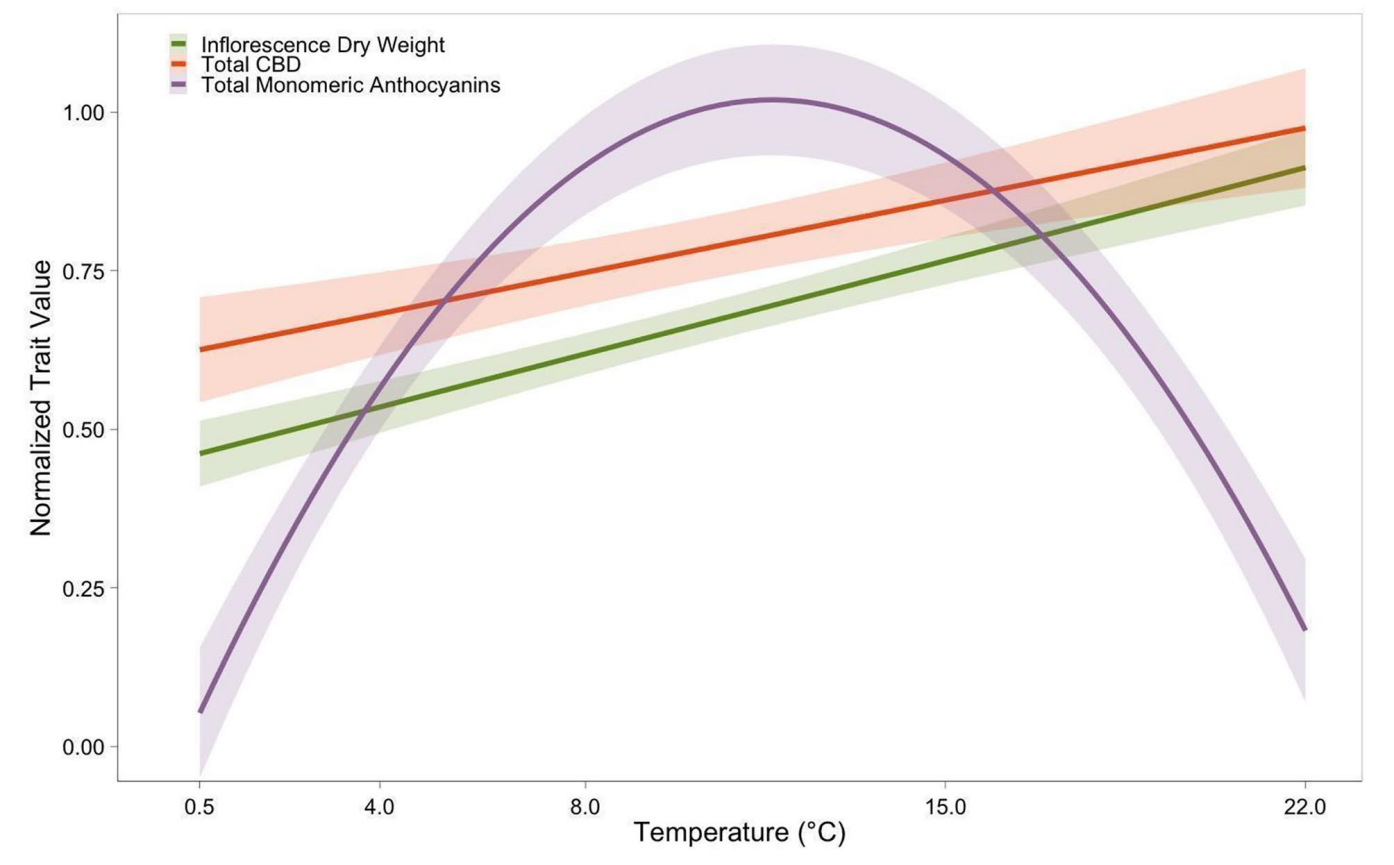
Normalized trait values for inflorescence dry weight (Green), total CBD (Orange), and total monomeric anthocyanins (Purple) for cannabis plants grown in various temperature treatments. The trendlines represent the overall modeled relationship between temperature and the three traits

The optimal temperature range for anthocyanin production was found to be distinct from that required for the inflorescence growth and cannabidiol synthesis (Fig. 6). Maximum anthocyanin levels at 8 °C and 15 °C, along with reduction at 0.5 °C and 22 °C, suggests distinct temperature-dependent regulatory pathways for anthocyanin biosynthesis in cannabis, separate from those influencing dry weight and CBD biosynthesis in the inflorescence (Fig. 6). Exposing cannabis plants to temperatures between 8 and 15 °C could result in the maximum anthocyanin concentration per gram of dried flower.

### Temperature stimuli influence anthocyanin accumulation in cannabis

Two fluctuating temperature treatments were introduced in the second replication of the experiment, with average temperatures of approximately 8 °C and 15 °C. These were simulated by fluctuating between 4 °C and 22 °C to investigate whether temperature stimuli or plant maturity has a greater effect on anthocyanin accumulation in the inflorescence of cannabis. Plants in the fluctuating treatments, exposed to an average temperature of approximately 8 °C (22 °C for 5 h & 4 °C for 19 h) and 15 °C (22 °C for 15 h & 4 °C for 9 h), exhibited inflorescence dry weight levels similar to plants exposed to constant temperatures of 8 °C and 15 °C (Fig. 1), while exhibiting total monomeric anthocyanin levels similar to plants exposed to constant 4 °C and 22 °C (Fig. 4). This indicates that fluctuating temperature conditions did not impede the maturity of the plants when compared to constant temperature conditions, suggesting that the reduction in anthocyanin accumulation in plants grown at 4 °C, relative to those at 8–15 °C, is likely due to temperature stimuli rather than a lack of growing degree days (GDD). The experimental groups subjected to fluctuating temperatures between 4 °C and 22 °C showed no significant differences in total anthocyanin concentration compared to plants cultivated at constant 4–22 °C. However, plants in the fluctuating temperature treatments did show significant differences when compared to those subjected to constant 8 °C and 15 °C conditions (Fig. 4). These results revealed that the observed effect of increased anthocyanin accumulation in the inflorescence of cannabis at cold temperatures in a range from 0.5 to 22 °C is attributable to temperature stimuli rather than plant maturity. Future work could consider how length of exposure to mild temperatures (8–15 °C) influences anthocyanin accumulation, such that plants can be maximized for growth (22 °C) and treated to increase anthocyanin concentration.

### Strong correlation between visual scores and calculated total monomeric anthocyanins

The strong and positive correlation observed between our visual score and total monomeric anthocyanins shows promise for an affordable and efficient visual assessment of anthocyanin accumulation in cannabis, eliminating the necessity for specialized equipment or costly instruments. Developing a protocol for quick and accurate phenotyping of purple pigmented cannabis cultivars, along with predictive assessments of anthocyanin concentration, will offer significant utility to breeders, farmers, and researchers. The positive correlation (0.7–0.8) observed between our visual ratings and the values obtained via spectrophotometry indicates potential for establishing such a protocol. Visual scores, when compared to spectroscopy results, were shown to have inflated values. The visual anthocyanin score may be somewhat inflated due to the limitation of visually assessing anthocyanins, which predominantly exist in the epidermal layer of plants. This method solely evaluates the exterior appearance of the plant, neglecting the anthocyanin concentration present throughout the entire plant. Additionally, it should be acknowledged that the correlation between visual scores and spectroscopy readings may become less accurate as the plant starts to senesce, highlighting the importance of assessing plants at equivalent stages of maturity.

## Conclusions

Our results demonstrate how anthocyanin accumulation, inflorescence dry weight, and CBD concentration in cannabis respond to cold temperatures in growth chamber conditions. Metabolic processes persisted at 4 °C, suggesting that the base temperature for cannabis, in growth chamber conditions, is lower than what has been previously documented. Our findings suggest that there are distinct physiological mechanisms governing anthocyanin accumulation when compared to those regulating inflorescence dry weight and the synthesis of CBD in cannabis inflorescences. While increasing temperatures may promote inflorescence dry weight and CBD production due to enhanced metabolic activity, they may also suppress anthocyanin production. Depending on the overall goal, maximizing one or a combination of these traits may be desired. Understanding the plants’ response to various temperature stimuli allows controlled-environment growers to manipulate their environment to optimize these traits, while also equipping outdoor growers with insights to effectively oversee their operations, such as determining optimal harvest times or cultivar selection.

## Supplementary Information

Below is the link to the electronic supplementary material.

Supplementary Material 1

## Data Availability

All collected data is available in Supplementary Table S1.

## References

[CR1] Akyüz FA, Ransom JK. Growing degree day calculation method comparison between two methods. in the Northern Edge of the US Corn Belt; 2015.

[CR3] Bassolino L, Zhang Y, Schoonbeek HJ, Kiferle C, Perata P, Martin C. Accumulation of anthocyanins in tomato skin extends shelf life. New Phytol. 2013;200(3):650–5. 10.1111/nph.12524. 24102530 10.1111/nph.12524

[CR2] Bassolino L, Fulvio F, Pastore C, Pasini F, Toschi G, Filippetti T, I., Paris R. When cannabis sativa L. Turns purple: biosynthesis and accumulation of anthocyanins. Antioxidants. 2023;12(7). 10.3390/antiox12071393. 10.3390/antiox12071393 PMC10376404 37507932

[CR4] Bates D, Mächler M, Bolker B, Walker S. Fitting linear Mixed-Effects models using lme4. J Stat Softw. 2015;67(1):1–48. 10.18637/jss.v067.i01.

[CR5] Bernstein N, Gorelick J, Zerahia R, Koch S. Impact of N, P, K, and humic acid supplementation on the chemical profile of medical cannabis (Cannabis sativa L). Front Plant Sci. 2019;10. 10.3389/fpls.2019.00736. 10.3389/fpls.2019.00736 PMC6589925 31263470

[CR6] Bonhomme R. (2000). Bases and limits to using degree.day units. In Eur J Agron (13). www.elsevier.com/locate/eja

[CR7] Boucher R, Germain H, Desgagné-Penix I. (2025). Exploring the Lesser-Known Bioactive Natural Products of Plant Species of the Genus Cannabis L.: Alkaloids, Phenolic Compounds, and Their Therapeutic Potential. In Plants (Vol. 14, Issue 9). Multidisciplinary Digital Publishing Institute (MDPI). 10.3390/plants14091372 10.3390/plants14091372 PMC12073235 40364401

[CR8] Chandra S, Lata H, Khan IA, ElSohly MA. Temperature response of photosynthesis in different drug and fiber varieties of cannabis sativa L. Physiol Mol Biology Plants. 2011;17(3):297–303. 10.1007/s12298-011-0068-4. 10.1007/s12298-011-0068-4 PMC3550580 23573022

[CR9] Chaves-Silva S, Santos AL, dos, Chalfun-Júnior A, Zhao J, Peres LEP, Benedito VA. Understanding the genetic regulation of anthocyanin biosynthesis in plants– Tools for breeding purple varieties of fruits and vegetables. Phytochemistry. Volume 153. Elsevier Ltd; 2018. pp. 11–27. 10.1016/j.phytochem.2018.05.013. 10.1016/j.phytochem.2018.05.013 29803860

[CR10] Choi KH, Lee HA, Park MH, Han JS. Cyanidin-3-rutinoside increases glucose uptake by activating the PI3K/Akt pathway in 3T3-L1 adipocytes. Environ Toxicol Pharmacol. 2017;54:1–6. 10.1016/j.etap.2017.06.007. 28667861 10.1016/j.etap.2017.06.007

[CR11] Choi KH, Park MH, Lee HA, Han JS. Cyanidin-3-rutinoside protects INS-1 pancreatic β cells against high glucose-induced glucotoxicity by apoptosis. Z Fur Naturforschung - Sect C J Biosci. 2018;73(7–8):281–9. 10.1515/znc-2017-0172. 10.1515/znc-2017-0172 29924740

[CR39] R Core Team. (2021). R: A language and environment for statistical computing (Version 4.1.1). R Foundation for Statistical Computing. https://www.R-project.org/

[CR12] Feng R, Ni HM, Wang SY, Tourkova IL, Shurin MR, Harada H, Yin XM. Cyanidin-3-rutinoside, a natural polyphenol antioxidant, selectively kills leukemic cells by induction of oxidative stress. J Biol Chem. 2007;282(18):13468–76. 10.1074/jbc.M610616200. 17360708 10.1074/jbc.M610616200

[CR13] Fox J, Weisberg S. An R companion to applied regression, third edition. Thousand Oaks CA: Sage; 2019. https://socialsciences.mcmaster.ca/jfox/Books/Companion/.

[CR14] Gagalova KK, Yan Y, Wang S, Matzat T, Castellarin SD, Birol I, Edwards D, Schuetz M. Leaf pigmentation in cannabis sativa: characterization of anthocyanin biosynthesis in colorful cannabis varieties. Plant Direct. 2024;8(11). 10.1002/pld3.70016. 10.1002/pld3.70016 PMC11588432 39600728

[CR15] He Q, Ren Y, Zhao W, Li R, Zhang L. Low temperature promotes anthocyanin biosynthesis and related gene expression in the seedlings of purple head Chinese cabbage (Brassica Rapa l). Genes. 2020;11(1). 10.3390/genes11010081. 10.3390/genes11010081 PMC7017278 31936856

[CR16] He R, Wei J, Zhang J, Tan X, Li Y, Gao M, Liu H. Supplemental blue light frequencies improve ripening and nutritional qualities of tomato fruits. Front Plant Sci. 2022;13. 10.3389/fpls.2022.888976. 10.3389/fpls.2022.888976 PMC9218689 35755648

[CR17] Huebner DS, Batarshin M, Beck S, König L, Mewis I, Ulrichs C. Influence of different UV spectra and intensities on yield and quality of cannabis inflorescences. Front Plant Sci. 2024;15. 10.3389/fpls.2024.1480876. 10.3389/fpls.2024.1480876 PMC11685020 39741668

[CR18] Jin D, Jin S, Chen J. Cannabis indoor growing conditions, management practices, and Post-Harvest treatment: A review. Am J Plant Sci. 2019;10(06):925–46. 10.4236/ajps.2019.106067.

[CR19] Jin HX, Jiang M, Yang JF, Wu ZH, Ma LL, Wang CC, Liang C, Ning XY, Ge LF, Chen S. A survey of enhanced cold tolerance and Low-Temperature-Induced anthocyanin accumulation in a novel Zoysia Japonica biotype. Plants. 2022;11(3). 10.3390/plants11030429. 10.3390/plants11030429 PMC8839389 35161412

[CR20] Kaur S, Tiwari V, Kumari A, Chaudhary E, Sharma A, Ali U, Garg M. Protective and defensive role of anthocyanins under plant abiotic and biotic stresses: an emerging application in sustainable agriculture. J Biotechnol (Vol. 2023;361:12–29. 10.1016/j.jbiotec.2022.11.009. Elsevier B.V. 10.1016/j.jbiotec.2022.11.009 36414125

[CR21] Khoo HE, Azlan A, Tang ST, Lim SM. (2017). Anthocyanidins and anthocyanins: Colored pigments as food, pharmaceutical ingredients, and the potential health benefits. In Food and Nutrition Research (Vol. 61). Swedish Nutrition Foundation. 10.1080/16546628.2017.1361779 10.1080/16546628.2017.1361779 PMC5613902 28970777

[CR22] Kongthitilerd P, Thilavech T, Marnpae M, Rong W, Yao S, Adisakwattana S, Cheng H, Suantawee T. Cyanidin-3-rutinoside stimulated insulin secretion through activation of L-type voltage-dependent Ca2 + channels and the PLC-IP3 pathway in pancreatic β-cells. Biomed Pharmacotherapy. 2022;146. 10.1016/j.biopha.2021.112494. 10.1016/j.biopha.2021.112494 34891116

[CR23] Kotiranta S, Sarka A, Kotilainen T, Palonen P. Decreasing R:FR ratio in a grow light spectrum increases inflorescence yield but decreases plant specialized metabolite concentrations in cannabis sativa. Environ Exp Bot. 2025;229. 10.1016/j.envexpbot.2024.106059.

[CR24] Krga I, Milenkovic D. Anthocyanins: from sources and bioavailability to Cardiovascular-Health benefits and molecular mechanisms of action. J Agric Food Chem. 2019;67(7):1771–83. 10.1021/acs.jafc.8b06737. 30698008 10.1021/acs.jafc.8b06737

[CR25] Lee J, Durst RW, Wrolstad RE, Barnes KW, Eisele T, Giusti MM, Haché J, Hofsommer H, Koswig S, Krueger DA, Kupina S, Martin SK, Martinsen BK, Miller TC, Paquette F, Ryabkova A, Skrede,G, Trenn,U, Wightman JD. (2005). Determination of Total Monomeric Anthocyanin Pigment Content of Fruit Juices, Beverages, Natural Colorants, and Wines by the pH Differential Method: Collaborative Study. https://academic.oup.com/jaoac/article/88/5/1269/5657437 16385975

[CR26] Leng X, Li C, Wang P, Ren Y, Chen J, Liu G, Hakeem A, Liu Y, Shi X, Hou T, Haider MS, Liu G, Fang J. The transcription factor VvMYB44-1 plays a role in reducing grapevine anthocyanin biosynthesis at high temperature. Plant Physiol. 2025;197(2):kiae657. 10.1093/plphys/kiae657. 39661410 10.1093/plphys/kiae657

[CR27] Lenth R. (2022). emmeans: Estimated marginal means, aka least-squares means. R package version 1.7. 2.

[CR28] Liu Y, Lin-Wang K, Espley Rv, Wang L, Li Y, Liu Z, Zhou P, Zeng L, Zhang X, Zhang J, Allan AC. StMYB44 negatively regulates anthocyanin biosynthesis at high temperatures in tuber flesh of potato. J Exp Bot. 2019;70(15):3809–24. 10.1093/jxb/erz194. 31020330 10.1093/jxb/erz194 PMC6685667

[CR29] Mattioli R, Francioso A, Mosca L, Silva P. (2020). Anthocyanins: A Comprehensive Review of Their Chemical Properties and Health Effects on Cardiovascular and Neurodegenerative Diseases. In Molecules (Vol. 25, Issue 17). MDPI AG. 10.3390/molecules25173809 10.3390/molecules25173809 PMC7504512 32825684

[CR30] McMaster GS, Wilhelm W, Wilhelm WW. (1997). Growing degree-days: one equation, two interpretations Growing degree-days: one equation, two interpretations AGRICULTURAL AND FOREST METEOROLOGY Growing degree-days: one equation, two interpretations. In Agricultural and Forest Meteorology (Vol. 87). https://www.digitalcommons.unl.edu/usdaarsfacpubhttps://www.digitalcommons.unl.edu/usdaarsfacpub

[CR31] Millard SP. (2014). EnvStats, an R Package for Environmental Statistics. In Wiley StatsRef: Statistics Reference Online (eds N. Balakrishnan, T. Colton, B. Everitt, W. Piegorsch, F. Ruggeri and J.L. Teugels). 10.1002/9781118445112.stat07181

[CR32] Naing AH, Kim CK. Abiotic stress-induced anthocyanins in plants: their role in tolerance to abiotic stresses. Physiologia plantarum. Volume 172. Blackwell Publishing Ltd; 2021. pp. 1711–23. 10.1111/ppl.13373. 10.1111/ppl.13373 33605458

[CR33] Oliveira H, Fernandes A, Brás NF, Mateus N, de Freitas V, Fernandes I. (2020). Anthocyanins as antidiabetic agents—in vitro and in silico approaches of preventive and therapeutic effects. In Molecules (Vol. 25, Issue 17). MDPI AG. 10.3390/molecules25173813 10.3390/molecules25173813 PMC7504281 32825758

[CR34] Oswald IWH, Paryani TR, Sosa ME, Ojeda MA, Altenbernd MR, Grandy JJ, Shafer NS, Ngo K, Peat JR, Melshenker BG, Skelly I, Koby KA, Page MFZ, Martin TJ. Minor, nonterpenoid volatile compounds drive the aroma differences of exotic cannabis. ACS Omega. 2023;8(42):39203–16. 10.1021/acsomega.3c04496. 37901519 10.1021/acsomega.3c04496 PMC10601067

[CR35] Pacheco SM, Soares MSP, Gutierres JM, Gerzson MFB, Carvalho FB, Azambuja JH, Schetinger MRC, Stefanello FM, Spanevello RM. Anthocyanins as a potential Pharmacological agent to manage memory deficit, oxidative stress and alterations in ion pump activity induced by experimental sporadic dementia of alzheimer’s type. J Nutr Biochem. 2018;56:193–204. 10.1016/j.jnutbio.2018.02.014. 29587242 10.1016/j.jnutbio.2018.02.014

[CR36] Popović D, Kocić G, Katić V, Jović Z, Zarubica A, Janković Veličković L, Nikolić V, Jović A, Kundalić B, Rakić V, Ulrih NP, Skrt M, Sokolović D, Dinić L, Stojanović M, Milosavljević A, Veličković F, Sokolović D. Protective effects of anthocyanins from Bilberry extract in rats exposed to nephrotoxic effects of carbon tetrachloride. Chemico-Biol Interact. 2019;304:61–72. 10.1016/j.cbi.2019.02.022. 10.1016/j.cbi.2019.02.022 30825423

[CR37] Pramananda V, Hadyan Fityay TA, Misran E. (2021). Anthocyanin as natural dye in DSSC fabrication: A review. IOP Conference Series: Materials Science and Engineering, 1122(1), 012104. 10.1088/1757-899x/1122/1/012104

[CR38] Punja ZK, Sutton DB, Kim T. Glandular trichome development, morphology, and maturation are influenced by plant age and genotype in high THC-containing cannabis (Cannabis sativa L.) inflorescences. J Cannabis Res. 2023;5(1). 10.1186/s42238-023-00178-9. 10.1186/s42238-023-00178-9 PMC10071647 37016398

[CR40] Shen B, Wu H, Xie X, Zhao B, Chen P, Ao D, Pan H, Lin B. Comparative transcriptomic analyses of anthocyanin biosynthesis genes in eggplant under low temperature and weak light. Plants. 2025;14(3). 10.3390/plants14030478. 10.3390/plants14030478 PMC11819703 39943040

[CR41] Sikora V, Berenji J, Latković D. Influence of agroclimatic conditions on content of main cannabinoids in industrial hemp (Cannabis sativa L). Genetika. 2011;43(3):449–56. 10.2298/GENSR1103449S.

[CR42] Singh S, Gaikwad KK, Lee YS. (2018). Anthocyanin– A Natural Dye for Smart Food Packaging Systems. KOREAN JOURNAL OF PACKAGING SCIENCE AND TECHNOLOGY, 24(3), 167–180. 10.20909/kopast.2018.24.3.167

[CR43] Smeriglio A, Barreca D, Bellocco E, Trombetta D. Chemistry, Pharmacology and health benefits of anthocyanins. Phytotherapy research. John Wiley and Sons Ltd; 2016. pp. 1265–86. 10.1002/ptr.5642. 10.1002/ptr.5642 27221033

[CR44] Thilavech T, Abeywardena MY, Adams M, Dallimore J, Adisakwattana S. Naturally occurring anthocyanin cyanidin-3-rutinoside possesses inherent vasorelaxant actions and prevents methylglyoxal-induced vascular dysfunction in rat aorta and mesenteric arterial bed. Biomed Pharmacotherapy. 2017;95:1251–9. 10.1016/j.biopha.2017.09.053. 10.1016/j.biopha.2017.09.053 28938516

[CR45] Thilavech T, Abeywardena MY, Dallimore J, Adams M, Adisakwattana S. Cyanidin-3-rutinoside alleviates methylglyoxal-induced cardiovascular abnormalities in the rats. J Funct Foods. 2018;49:258–66. 10.1016/j.jff.2018.08.034.

[CR46] U.S. Department of Agriculture. Establishment of a domestic hemp production program: final rule. Fed Reg. 2021;86(11):5596–712. https://www.federalregister.gov/documents/2021/01/19/2021-00967/establishment-of-a-domestic-hemp-production-program.

[CR47] Wickham H. (2009). Ggplot2, elegant graphics for data Analysis,177–82. 10.1007/978-0-387-98141-3_10

[CR48] Zhang Y, Butelli E, de Stefano R, Schoonbeek HJ, Magusin A, Pagliarani C, Wellner N, Hill L, Orzaez D, Granell A, Jones JDG, Martin C. Anthocyanins double the shelf life of tomatoes by delaying overripening and reducing susceptibility to Gray mold. Curr Biol. 2013;23(12):1094–100. 10.1016/j.cub.2013.04.072. 23707429 10.1016/j.cub.2013.04.072 PMC3688073

[CR49] Zoratti L, Karppinen K, Escobar AL, Häggman H, Jaakola L. (2014). Light-controlled flavonoid biosynthesis in fruits. In Frontiers in Plant Science (Vol. 5, Issue OCT). Frontiers Media S.A. 10.3389/fpls.2014.00534 10.3389/fpls.2014.00534 PMC4191440 25346743

