# Supplementary material for: Anthocyanin accumulation, inflorescence dry weight and total cannabidiol content have different temperature optima in Cannabis sativa
Source: J Cannabis Res. 2025 Jul 29;7:51. doi: 10.1186/s42238-025-00311-w (PMC12309197; doi:10.1186/s42238-025-00311-w)
Supplement: Supplementary file 1 — Supplementary Material 1 [file 42238_2025_311_MOESM1_ESM.docx]

**Supplementary Figures**

**
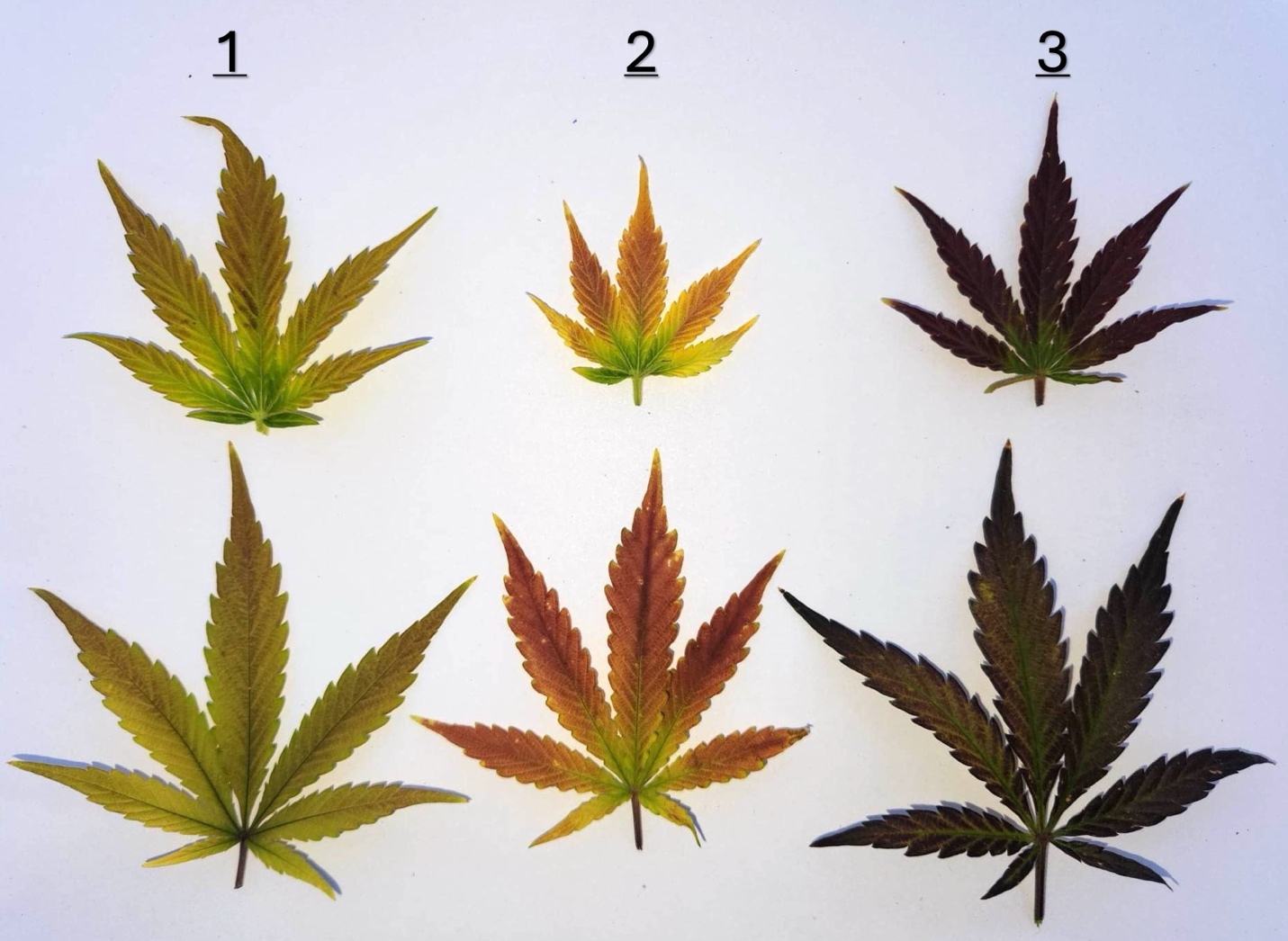
**

**Figure S1.** Leaf tissue representations of the color grade used to obtain visual scores of plants subject to temperature treatments. Color hue descriptions are as follows: 1 (lilac/pink), 2 (magenta/reddish), and 3 (purple/dark purple). The total anthocyanin coverage (%) across the entire plant was also quantified. These combined scores facilitated the calculation of the total anthocyanin score (TAS) for each plant.


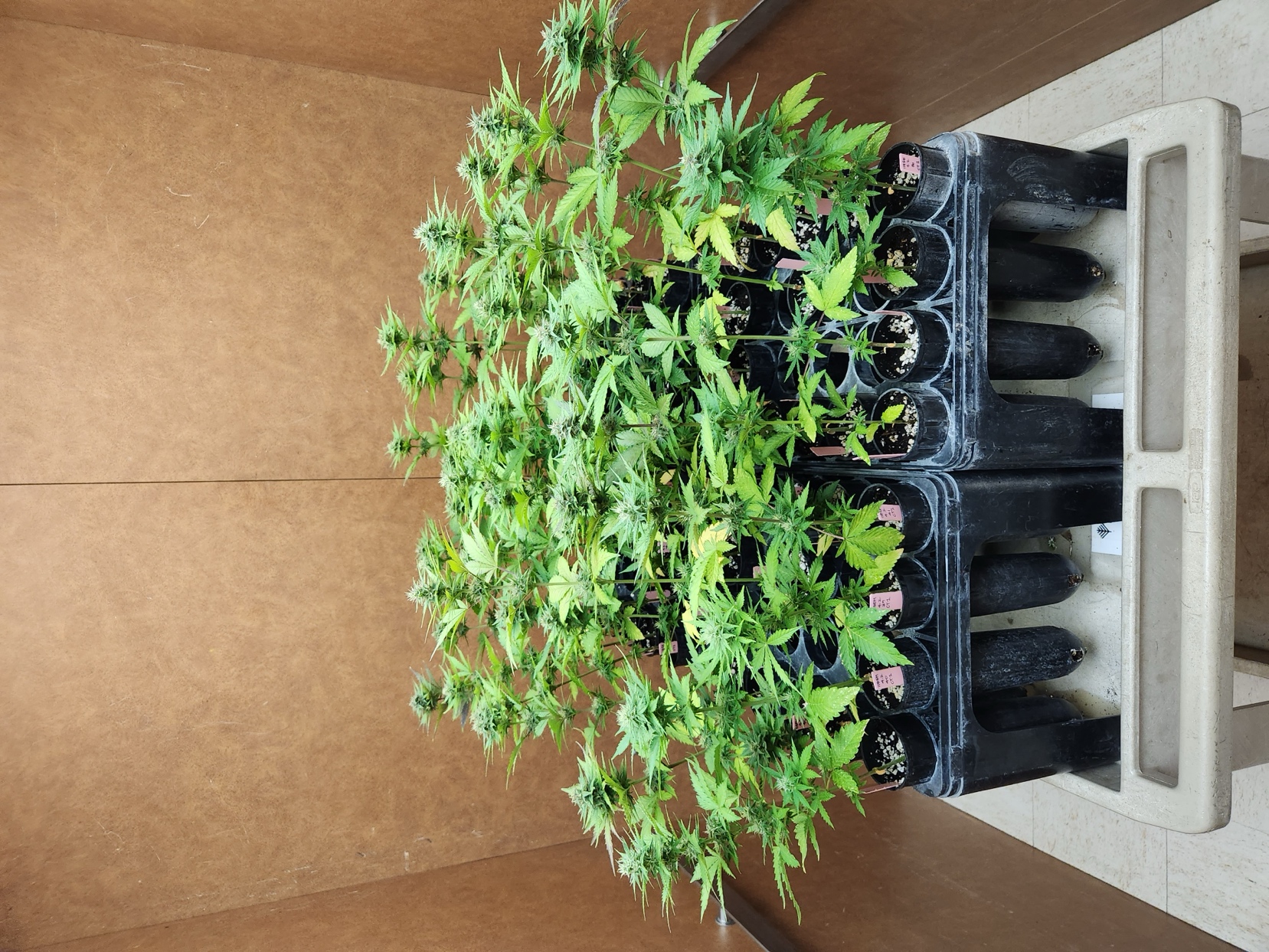


**Figure S2.** Randomized day-neutral cultivars before entering the first replication of the temperature treatments. Each plant grown for 42 days (15 days after flower initiation). Plants showed no signs of anthocyanin accumulation prior to entering their respective treatments.

**Supplemental Tables**

**Table S1.** Raw data from two replicated temperature treatment studies. Condition represents whether the environmental temperature was constant or fluctuating. Data was collected on the following traits: total anthocyanin score (TAS), total dry weight (g) (TDW), total monomeric anthocyanins (TMA) evaluated as cyanidin-3-glucoside equivalents (mg/L), and total CBD (%) (TCBD).

| **Plant ID** | **Repeat** | **Condition** | **Temperature** | **TAS** | **TDW** | **TMA** | **TCBD** |
| --- | --- | --- | --- | --- | --- | --- | --- |
| **16** | Repeat 1 | Const | 0.5 | 20 | 1.18 | 3 | 5.84 |
| **17** | Repeat 1 | Const | 0.5 | 10 | 1.52 | 5 | 1.84 |
| **19** | Repeat 1 | Const | 0.5 | 3 | 1.24 | 2 | 2.66 |
| **20** | Repeat 1 | Const | 0.5 | 20 | 1.43 | 6 | 3.4 |
| **22** | Repeat 1 | Const | 0.5 | 15 | 1.37 | 3 | 4.63 |
| **25** | Repeat 1 | Const | 0.5 | 15 | 1.59 | 4 | 3.24 |
| **26** | Repeat 1 | Const | 0.5 | 10 | 1.21 | 1 | 4.97 |
| **32** | Repeat 1 | Const | 0.5 | 3 | 1.05 | 0 | 3.01 |
| **36** | Repeat 1 | Const | 0.5 | 6 | 1.26 | 0 | 3.63 |
| **43** | Repeat 1 | Const | 0.5 | 6 | 0.83 | 2 | 2.53 |
| **58** | Repeat 1 | Const | 0.5 | 4 | 0.93 | 3 | 2.37 |
| **64** | Repeat 1 | Const | 0.5 | 2 | 0.46 | 3 | 1.28 |
| **78** | Repeat 1 | Const | 0.5 | 2 | 0.63 | 1 | 1.97 |
| **79** | Repeat 1 | Const | 0.5 | 2 | 0.38 | 2 | 2.63 |
| **85** | Repeat 1 | Const | 0.5 | 2 | 0.56 | 0 | 1.76 |
| **4** | Repeat 1 | Const | 4 | 160 | 1.6 | 32 | 6.57 |
| **10** | Repeat 1 | Const | 4 | 375 | 1.62 | 37 | 2.8 |
| **27** | Repeat 1 | Const | 4 | 350 | 1.76 | 88 | 6.05 |
| **42** | Repeat 1 | Const | 4 | 325 | 1.72 | 45 | 3.13 |
| **44** | Repeat 1 | Const | 4 | 325 | 1.32 | 32 | 4.72 |
| **48** | Repeat 1 | Const | 4 | 375 | 1.57 | 52 | 5.17 |
| **50** | Repeat 1 | Const | 4 | 250 | 1.2 | 46 | 2.66 |
| **51** | Repeat 1 | Const | 4 | 375 | 1.23 | 65 | 2.55 |
| **52** | Repeat 1 | Const | 4 | 450 | 2.07 | 61 | 6.56 |
| **54** | Repeat 1 | Const | 4 | 250 | 1.29 | 32 | 2.13 |
| **56** | Repeat 1 | Const | 4 | 375 | 1.59 | 47 | 3.79 |
| **57** | Repeat 1 | Const | 4 | 200 | 1.71 | 34 | 4.63 |
| **59** | Repeat 1 | Const | 4 | 375 | 1.74 | 65 | 5.22 |
| **63** | Repeat 1 | Const | 4 | 450 | 0.48 | 24 | 3.65 |
| **66** | Repeat 1 | Const | 4 | 480 | 0.8 | 65 | 2 |
| **15** | Repeat 1 | Const | 8 | 540 | 1.67 | 40 | 3.67 |
| **23** | Repeat 1 | Const | 8 | 540 | 1.84 | 67 | 2.87 |
| **29** | Repeat 1 | Const | 8 | 570 | 1.55 | 71 | 4.53 |
| **33** | Repeat 1 | Const | 8 | 510 | 1.82 | 50 | 4.68 |
| **34** | Repeat 1 | Const | 8 | 570 | 1.87 | 50 | 6.24 |
| **37** | Repeat 1 | Const | 8 | 510 | 1.59 | 50 | 2.49 |
| **38** | Repeat 1 | Const | 8 | 540 | 1.81 | 22 | 3.7 |
| **39** | Repeat 1 | Const | 8 | 540 | 1.59 | 28 | 3.16 |
| **40** | Repeat 1 | Const | 8 | 510 | 1.88 | 65 | 2.47 |
| **41** | Repeat 1 | Const | 8 | 570 | 1.78 | 81 | 4.1 |
| **46** | Repeat 1 | Const | 8 | 540 | 2.16 | 51 | 3.7 |
| **67** | Repeat 1 | Const | 8 | 540 | 0.93 | 63 | 2.53 |
| **80** | Repeat 1 | Const | 8 | 570 | 0.67 | 121 | 3.33 |
| **81** | Repeat 1 | Const | 8 | 570 | 0.92 | 102 | 3.24 |
| **82** | Repeat 1 | Const | 8 | 570 | 0.88 | 99 | 3.14 |
| **2** | Repeat 1 | Const | 15 | 570 | 2.39 | 35 | 4.16 |
| **3** | Repeat 1 | Const | 15 | 540 | 2.37 | 62 | 10.59 |
| **5** | Repeat 1 | Const | 15 | 540 | 1.86 | 72 | 5.92 |
| **7** | Repeat 1 | Const | 15 | 570 | 2.61 | 26 | 3.49 |
| **9** | Repeat 1 | Const | 15 | 570 | 2.47 | 85 | 4.79 |
| **11** | Repeat 1 | Const | 15 | 570 | 1.94 | 43 | 2.65 |
| **13** | Repeat 1 | Const | 15 | 510 | 2.12 | 20 | 5.06 |
| **14** | Repeat 1 | Const | 15 | 540 | 2.34 | 38 | 4.66 |
| **24** | Repeat 1 | Const | 15 | 570 | 2.5 | 59 | 8.86 |
| **31** | Repeat 1 | Const | 15 | 540 | 1.69 | 50 | 4.53 |
| **47** | Repeat 1 | Const | 15 | 540 | 2.18 | 86 | 7.6 |
| **60** | Repeat 1 | Const | 15 | 570 | 2.31 | 94 | 9.23 |
| **61** | Repeat 1 | Const | 15 | 540 | 1.05 | 46 | 5.46 |
| **76** | Repeat 1 | Const | 15 | 570 | 1.36 | 31 | 4.08 |
| **77** | Repeat 1 | Const | 15 | 540 | 0.99 | 92 | 5.27 |
| **1** | Repeat 1 | Const | 22 | 375 | 3.26 | 8 | 5.75 |
| **6** | Repeat 1 | Const | 22 | 425 | 2.88 | 28 | 7.16 |
| **8** | Repeat 1 | Const | 22 | 375 | 2.8 | 4 | 3.58 |
| **12** | Repeat 1 | Const | 22 | 400 | 2.39 | 27 | 3.37 |
| **18** | Repeat 1 | Const | 22 | 480 | 2.85 | 13 | 7.45 |
| **21** | Repeat 1 | Const | 22 | 425 | 3.05 | 8 | 8.97 |
| **28** | Repeat 1 | Const | 22 | 400 | 2.53 | 11 | 7.7 |
| **30** | Repeat 1 | Const | 22 | 320 | 2.27 | 14 | 4.04 |
| **35** | Repeat 1 | Const | 22 | 510 | 2.1 | 17 | 3.91 |
| **49** | Repeat 1 | Const | 22 | 260 | 3.17 | 0 | 6.6 |
| **53** | Repeat 1 | Const | 22 | 280 | 2.49 | 3 | 5.36 |
| **55** | Repeat 1 | Const | 22 | 300 | 2.75 | 8 | 6.28 |
| **62** | Repeat 1 | Const | 22 | 350 | 1.24 | 11 | 2.6 |
| **83** | Repeat 1 | Const | 22 | 70 | 0.93 | 3 | 4.44 |
| **86** | Repeat 1 | Const | 22 | 300 | 1.25 | 15 | 5.34 |
| **CT2 1** | Repeat 2 | Const | 0.5 | 60 | 0.89 | 4 | 3.73 |
| **CT2 10** | Repeat 2 | Const | 0.5 | 28 | 1.16 | 0 | 5.93 |
| **CT2 11** | Repeat 2 | Const | 0.5 | 40 | 1.18 | 4 | 6.97 |
| **CT2 12** | Repeat 2 | Const | 0.5 | 5 | 0.98 | 1 | 3.87 |
| **CT2 13** | Repeat 2 | Const | 0.5 | 40 | 1.01 | 3 | 3.96 |
| **CT2 2** | Repeat 2 | Const | 0.5 | 60 | 0.96 | 8 | 4.23 |
| **CT2 3** | Repeat 2 | Const | 0.5 | 0 | 0.94 | 0 | 4.74 |
| **CT2 4** | Repeat 2 | Const | 0.5 | 10 | 1.25 | 1 | 4.39 |
| **CT2 5** | Repeat 2 | Const | 0.5 | 10 | 0.91 | 0 | 5.58 |
| **CT2 6** | Repeat 2 | Const | 0.5 | 0 | 0.59 | 1 | 1.63 |
| **CT2 7** | Repeat 2 | Const | 0.5 | 60 | 0.57 | 2 | 2.04 |
| **CT2 8** | Repeat 2 | Const | 0.5 | 100 | 0.93 | 3 | 4.71 |
| **CT2 9** | Repeat 2 | Const | 0.5 | 10 | 1.16 | 0 | 2.89 |
| **CT2 14** | Repeat 2 | Const | 4 | 360 | 0.5 | 15 | 0.59 |
| **CT2 15** | Repeat 2 | Const | 4 | 330 | 1.13 | 20 | 3.39 |
| **CT2 16** | Repeat 2 | Const | 4 | 360 | 1.2 | 43 | 3.05 |
| **CT2 17** | Repeat 2 | Const | 4 | 390 | 1.37 | 36 | 2.35 |
| **CT2 18** | Repeat 2 | Const | 4 | 360 | 1.29 | 35 | 5.24 |
| **CT2 19** | Repeat 2 | Const | 4 | 75 | 0.36 | 3 | NA |
| **CT2 20** | Repeat 2 | Const | 4 | 300 | 1.27 | 18 | 2.85 |
| **CT2 21** | Repeat 2 | Const | 4 | 270 | 1.39 | 16 | 6.01 |
| **CT2 22** | Repeat 2 | Const | 4 | 300 | 1.02 | 14 | 1.73 |
| **CT2 23** | Repeat 2 | Const | 4 | 330 | 1.47 | 38 | 4.76 |
| **CT2 24** | Repeat 2 | Const | 4 | 360 | 1.3 | 28 | 3.35 |
| **CT2 25** | Repeat 2 | Const | 4 | 360 | 1.48 | 34 | 1.55 |
| **CT2 26** | Repeat 2 | Const | 4 | 420 | 1.15 | 30 | 2.15 |
| **CT2 27** | Repeat 2 | Const | 8 | 540 | 0.93 | 112 | 4.06 |
| **CT2 28** | Repeat 2 | Const | 8 | 360 | 0.72 | 23 | 1.02 |
| **CT2 29** | Repeat 2 | Const | 8 | 570 | 1.67 | 93 | NA |
| **CT2 30** | Repeat 2 | Const | 8 | 510 | 1.56 | 81 | 1.51 |
| **CT2 31** | Repeat 2 | Const | 8 | 510 | 0.94 | 57 | 1.66 |
| **CT2 32** | Repeat 2 | Const | 8 | 570 | 1.24 | 111 | 5.7 |
| **CT2 33** | Repeat 2 | Const | 8 | 540 | 1.33 | 72 | 5.56 |
| **CT2 34** | Repeat 2 | Const | 8 | 570 | 1.91 | 94 | 2.57 |
| **CT2 35** | Repeat 2 | Const | 8 | 540 | 1.18 | 90 | 1.93 |
| **CT2 36** | Repeat 2 | Const | 8 | 480 | 0.87 | 23 | NA |
| **CT2 37** | Repeat 2 | Const | 8 | 540 | 1.18 | 68 | 3.42 |
| **CT2 38** | Repeat 2 | Const | 8 | 510 | 1.24 | 81 | 3.47 |
| **CT2 39** | Repeat 2 | Const | 8 | 480 | 1.45 | 35 | 4.23 |
| **CT2 40** | Repeat 2 | Const | 15 | 540 | 1.79 | 80 | 4.59 |
| **CT2 41** | Repeat 2 | Const | 15 | 570 | 1.46 | 64 | 3.4 |
| **CT2 42** | Repeat 2 | Const | 15 | 510 | 1.5 | 34 | 2.3 |
| **CT2 43** | Repeat 2 | Const | 15 | 540 | 1.44 | 43 | 5.37 |
| **CT2 44** | Repeat 2 | Const | 15 | 480 | 1.43 | 31 | 1.97 |
| **CT2 45** | Repeat 2 | Const | 15 | 540 | 1.47 | 40 | 5.9 |
| **CT2 46** | Repeat 2 | Const | 15 | 540 | 1.59 | 88 | 8.2 |
| **CT2 47** | Repeat 2 | Const | 15 | 480 | 1.74 | 57 | 2.96 |
| **CT2 48** | Repeat 2 | Const | 15 | 510 | 1.29 | 45 | 2.56 |
| **CT2 49** | Repeat 2 | Const | 15 | 570 | 1.98 | 150 | 3.96 |
| **CT2 50** | Repeat 2 | Const | 15 | 480 | 1.57 | 35 | 5.17 |
| **CT2 51** | Repeat 2 | Const | 15 | 375 | 1.98 | 22 | 3.91 |
| **CT2 52** | Repeat 2 | Const | 15 | 540 | 1.62 | 88 | 3.08 |
| **CT2 79** | Repeat 2 | Const | 22 | 510 | 1.61 | 39 | 2.95 |
| **CT2 80** | Repeat 2 | Const | 22 | 150 | 1.25 | 5 | 5.81 |
| **CT2 81** | Repeat 2 | Const | 22 | 360 | 2 | 23 | 4.09 |
| **CT2 82** | Repeat 2 | Const | 22 | 420 | 1.76 | 51 | 7.29 |
| **CT2 83** | Repeat 2 | Const | 22 | 275 | 1.52 | 15 | 3.4 |
| **CT2 84** | Repeat 2 | Const | 22 | 250 | 1.73 | 0 | 2.13 |
| **CT2 85** | Repeat 2 | Const | 22 | 225 | 1.24 | 8 | 2.95 |
| **CT2 86** | Repeat 2 | Const | 22 | 480 | 2.23 | 32 | 8.07 |
| **CT2 87** | Repeat 2 | Const | 22 | 300 | 2.28 | 16 | 4.51 |
| **CT2 88** | Repeat 2 | Const | 22 | 160 | 1.54 | 9 | 6.94 |
| **CT2 89** | Repeat 2 | Const | 22 | 275 | 2.38 | 11 | 2.64 |
| **CT2 90** | Repeat 2 | Const | 22 | 180 | 1.12 | 15 | 4.76 |
| **CT2 91** | Repeat 2 | Const | 22 | 225 | 1.41 | 4 | 6.28 |
| **CT2 66** | Repeat 2 | Fluct | 7.75 | 390 | 1.4 | 49 | 3.22116 |
| **CT2 67** | Repeat 2 | Fluct | 7.75 | 420 | 1.44 | 37 | 3.53672 |
| **CT2 68** | Repeat 2 | Fluct | 7.75 | 360 | 1.41 | 23 | 4.58848 |
| **CT2 69** | Repeat 2 | Fluct | 7.75 | 250 | 1.46 | 18 | 4.85773 |
| **CT2 70** | Repeat 2 | Fluct | 7.75 | 360 | 1.37 | 27 | 5.22067 |
| **CT2 71** | Repeat 2 | Fluct | 7.75 | 360 | 1.14 | 28 | 2.70266 |
| **CT2 72** | Repeat 2 | Fluct | 7.75 | 100 | 0.56 | 7 | 1.14561 |
| **CT2 73** | Repeat 2 | Fluct | 7.75 | 250 | 1.42 | 16 | 6.66045 |
| **CT2 74** | Repeat 2 | Fluct | 7.75 | 140 | 1.06 | 12 | 3.34838 |
| **CT2 75** | Repeat 2 | Fluct | 7.75 | 175 | 1.45 | 37 | 3.12207 |
| **CT2 76** | Repeat 2 | Fluct | 7.75 | 330 | 1.26 | 33 | 5.80318 |
| **CT2 77** | Repeat 2 | Fluct | 7.75 | 360 | 1.14 | 28 | 5.95286 |
| **CT2 78** | Repeat 2 | Fluct | 7.75 | 225 | 1.07 | 20 | 2.04828 |
| **CT2 53** | Repeat 2 | Fluct | 15.25 | 480 | 2.33 | 44 | 4.01693 |
| **CT2 54** | Repeat 2 | Fluct | 15.25 | 450 | 2.2 | 33 | 2.52004 |
| **CT2 55** | Repeat 2 | Fluct | 15.25 | 375 | 1.42 | 33 | 7.22905 |
| **CT2 56** | Repeat 2 | Fluct | 15.25 | 450 | 1.24 | 50 | 5.42703 |
| **CT2 57** | Repeat 2 | Fluct | 15.25 | 350 | 1.67 | 24 | 3.85308 |
| **CT2 58** | Repeat 2 | Fluct | 15.25 | 360 | 1.12 | 29 | 6.58943 |
| **CT2 59** | Repeat 2 | Fluct | 15.25 | 220 | 1.07 | 6 | 2.61972 |
| **CT2 60** | Repeat 2 | Fluct | 15.25 | 300 | 0.81 | 11 | 2.36464 |
| **CT2 61** | Repeat 2 | Fluct | 15.25 | 450 | 2.06 | 30 | 5.17596 |
| **CT2 62** | Repeat 2 | Fluct | 15.25 | 450 | 1.52 | 40 | 5.22981 |
| **CT2 63** | Repeat 2 | Fluct | 15.25 | 480 | 1.44 | 46 | 3.23688 |
| **CT2 64** | Repeat 2 | Fluct | 15.25 | 420 | 1.71 | 43 | 4.99056 |
| **CT2 65** | Repeat 2 | Fluct | 15.25 | 420 | 1.52 | 36 | 8.01065 |

**Table S2.** Average values of total dry weight (TDW), total CBD (TCBD), total monomeric anthocyanins (TMA), and total anthocyanin score (TAS) for each temperature treatment across two repeated studies. Note: 8°C fluctuating and 15°C fluctuating treatments were only included in the second study.

| Treatment | Total Dry Weight (g) (TDW) | Total CBD (%)  (TCBD) | Total Monomeric Anthocyanins (mg/L) (TMA) | Total Anthocyanin Score (TAS) |
| --- | --- | --- | --- | --- |
| 0.5°C | 1.01 | 3.59 | 2 | 19 |
| 4°C | 1.31 | 3.65 | 26 | 333 |
| 8°C | 1.40 | 3.42 | 68 | 533 |
| 15°C | 1.82 | 4.99 | 58 | 534 |
| 22°C | 2.07 | 5.16 | 14 | 324 |
| 8°C fluctuating | 1.24 | 4.02 | 26 | 286 |
| 15°C fluctuating | 1.55 | 4.71 | 33 | 400 |
